# Binge Drinking among Economically Disadvantaged African American Older Adults with Diabetes

**DOI:** 10.3390/bs9090097

**Published:** 2019-09-11

**Authors:** Shervin Assari, James L. Smith, Mohammed Saqib, Mohsen Bazargan

**Affiliations:** 1 Departments of Family Medicine, College of Medicine, Charles R Drew University of Medicine and Science, 118th St, Los Angeles, CA 90059, USA; 2 Department of Health Behavior and Health Education, University of Michigan, University of Michigan, Ann Arbor, MI 48109, USA; 3 Departments of Family Medicine, University of California, Los Angeles (UCLA), Los Angeles, CA 90095, USA

**Keywords:** ethnic, ethnicity, ethnic groups, African American, black, older adults, diabetes, binge, drinking

## Abstract

*Purpose.* This study investigated the effect of demographic, socioeconomic, and psychological factors as well as the role of health determinants on alcohol consumption and binge drinking among economically disadvantaged African American older adults with type 2 diabetes mellites (T2DM). *Methods.* This survey recruited 231 African Americans who were older adults (age 65+ years) and had T2DM. Participants were selected from economically disadvantaged areas of South Los Angeles. A structured face-to-face interview was conducted to collect data on demographic factors, objective and subjective socioeconomic status (SES) including education and financial difficulty, living arrangement, marital status, health, and drinking behaviors (drinking and binge drinking). *Results.* Age, gender, living alone, pain, comorbid conditions, and smoking were associated with drinking/binge drinking. Male gender, pain, and being a smoker were associated with higher odds of drinking/binge drinking, while individuals with more comorbid medical conditions had lower odds of binge drinking. *Conclusion.* In economically constrained urban environments, gender, pain, and smoking but not age, SES, depression, and health may predict binge drinking for African American older adults with T2DM. African Americans older adult men with T2DM with comorbid pain should be screened for binge drinking.

## 1. Background

Type 2 diabetes mellites (T2DM) is more common and disabling in African Americans than White Americans [1]. African American patients with diabetes have worse disease management, self-care, worse glycemic control, and worse long-term outcomes [2]. As a result, there is a growing interest in understanding barriers that are associated with poor health outcomes of African Americans with T2DM [3]. There is also a tremendous interest in finding ways to enhance self-care and outcomes of African Americans with T2DM, particularly those who are economically challenged [4].

Drinking alcohol in general, and binge drinking in particular, are among the main behavioral barriers against successful T2DM self-care and disease management across populations [5]. As a result, there is a need to understand which factors increase the risk of drinking alcohol and binge drinking among individuals with T2DM [6]. Such information may help develop programs and interventions that reduce alcohol use of patients diagnosed with diabetes [7].

Very little information, however, exists on prevalence and correlates of drinking and binge drinking for African American older adults with T2DM in urban settings. This information would help inform alcohol treatment programs for African Americans patients with T2DM for whom binge drinking is a major barrier to achieving more favorable disease prognoses related to T2DM [6,7,8]. These become particularly relevant given high vulnerability of African Americans to alcohol use [9,10,11,12,13,14]. 

Overall, younger age is known to be a strong predictor of drinking and binge drinking, with younger individuals being at a higher risk [15]. Gender is also a major determinant of alcohol use and binge alcohol use disorder, with men being at a higher risk than women [15]. Some of these age and gender/sex differences are due to biological [16] and some others are due to socialization processes [17]. Women have a lower tolerance for alcohol due to a deficiency in alcohol metabolizing enzymes, which is partly due to sex hormones [18]. Older adults also have a lower metabolism of alcohol [19]. At the same time, given the traditional gender norms, the society better accepts men than women to drink frequently [17]. Social learning shapes results in considerable gender differences in drinking patterns [17].

In addition to demographic factors, socioeconomic status (SES) strongly influences alcohol use disorders. SES impacts all aspects of health including a wide range of health behaviors [20]. Low SES is a predictor of smoking [21] and drinking [22]. A high number of scholars have theorized how low SES operates as a risk factor for health risk behaviors such as alcohol binge drinking [23]. Alcohol use of sub-populations, however, is not similarly affected by SES indicators [24,25,26,27].

Both objective and subjective SES indicators may influence substance use of populations. Among various SES indicators, educational attainment and financial difficulty have been shown to shape health and health behaviors of populations [28,29]. Low SES (educational attainment and higher financial difficulty) contribute to the existing racial and ethnic health disparities that are experienced by the African American community [30,31]. In this view, one reason why alcohol use disorder is more common in African American communities is lower educational attainment and higher financial difficulty [32].

Another block of variables that may shape individuals’ drinking behaviors is their health indicators such as chronic medical conditions. For at least two reasons, we observe a link between chronic disease and drinking. First people who binge drink are at an increased risk of chronic medical conditions (CMCs) such as T2DM, heart disease, stroke, and some types of cancer [33]. Alternatively, people may quit drinking and other risk behaviors as a response to being diagnosed with a new CMC [34,35]. That means, CMCs and drinking may show positive [33] or negative [34,35] associations. These effects may cancel out the effect of each other. 

Loneliness is also a risk factor for alcohol use disorders. As many people drink in social gatherings [36], size of social network is a predictor of drinking behaviors [37]. Loneliness, and social isolation, are major problems in older adults including African American older adults [36]. Loneliness may be also associated with an increased risk of drinking, simply because living alone may be associated with higher levels of psychological distress (e.g., rumination of stressors) [38]. 

In addition, chronic physical pain is another correlate of alcohol use [39]. One hypothesis is that individuals experiencing chronic pain may use alcohol to cope with pain. Alcohol is an analgesic and brings amnesia and numbness, which may explain why some individuals with chronic pain self-medicate with using alcohol [39]. 

Finally, depression is shown to be associated with alcohol use disorders [40]. Given the very close link between depression and substance use disorder, a large proportion of patients with depression also have substance use disorder (e.g., dual diagnosis) [41]. The issue of dual diagnosis is also well described for African Americans [42]. In fact, some people may use alcohol as a coping mechanism to deal with their psychological pain, particularly those from mood problems, depression, and anxiety [43]. 

## 2. Aims

The current study explored the role of demographic factors, SES, and health determinants in drinking and alcohol binge drinking in African American older adults diagnosed with T2DM. 

## 3. Methods

### 3.1. Design & Setting

This cross-sectional study included a comprehensive home-based evaluation of medications in addition to a structured face-to-face interview of a community sample of African American older adults. This household survey was performed in South Los Angeles 2015-208 [44,45,46]. The study collected extensive data on demographic factors, SES, healthcare access, health status, smoking, drinking, and binge drinking. 

### 3.2. Institutional Review Board (IRB)

All the participants provided a signed informed consent. The protocol of the current investigation was approved by the IRB at the Charles R. Drew University of Medicine and Science (CDU University, Approval No.: CDU IRB# 14-12-2450-05). 

### 3.3. Participants & Sampling

The original study recruited a convenient sample of African American older adults who were at least 55 years old (n = 740). This study, however, limits its sample to participants who were non-institutionalized, Black/ African American, aged 65+ years, and had T2DM (n = 230). Most of the participants were recruited from predominantly African American housing units, projects, and senior residential centers, all located in South Los Angeles. No participants were selected from nursing facilities. Another exclusion criterion was enrollment in any other clinical trials. In addition, individuals with considerable cognitive deficits were excluded from this study. 

### 3.4. Study Measures

#### Independent Variables

*Demographic Factors.* Age (years) and gender (1 male, 0 females) were demographic covariates, treated as continuous and dichotomous variables, respectively. 

*Socioeconomic Status (SES).* This study included two SES indicators. Educational attainment was the only measure of objective SES. Educational attainment was a continuous measure (years of schooling). A higher score for our education variables meant more years of education (higher objective SES). Financial difficulty was the subjective SES indicator. Self-reported (perceived) financial difficulty was measured using three items questions that were in line with the Pearlin’s list of main chronic financial difficulties that are being experienced by the low SES individuals [47,48,49]. These items cover whether the participant felt they had money enough to meet most essential needs. Responses were on a 5 point scale, responses scale ranging from ‘never’ to ‘always’. A total score was calculated with a high score reflecting more financial difficulty. Cronbach alpha of the measure in this study was excellent (alpha = 0.923).

*Living Arrangement.* Participants’ living arrangement was a single item. We asked participants whether they are living alone or there is any other member of the family such as partner or spouse who is accompanying them. Living arrangement indicates loneliness, which is a strong determinant of health in older adults. Loneliness and living alone is shown to have a large effect in alcohol use [36,37].

*Marital Status.* Participants’ marital status was a dichotomous variable: 1 married vs. 0 any other situation such as divorced, never married, widow/widower, etc.

*Health Insurance Status.* Participants were asked if they have health insurance. Any type of health insurance including private, federal, or state level were considered. Health insurance was a dichotomous variable that disregarded its type. We did not include health insurance in the multivariable analysis because more than 95% of our sample had health care coverage. This variable did not have a good distribution to show any potential effect on our outcomes.

*Comorbid Medical Conditions (CMC).* Participants were asked about their 10 CMCs. The conditions included thyroid disorder, hypertension, heart disease, asthma, osteoarthritis, chronic obstructive pulmonary disease, rheumatoid arthritis, cancer, gastrointestinal disease, and lipid disorder/hypercholesterolemia. Self-reported CMC is valid [50,51,52].

*Self-Rated Health (SRH).* SRH was a single-item health measure that ranged from 1 to 5, reflecting “very good,” “good,” “fair,” “bad,” and “very bad” health. A higher score indicates worse health. Poor SRH is a strong predictor of mortality and morbidity [53,54,55,56].

*Sick Days.* % sick days was measured using the following single item: “In the past 12 months, how frequently have you been sick?”. Responses were (1) always, (2) most of the time, (3) sometimes, (4) rarely, and (5) never. This item was treated as a continuous measure with a range from 1 to 5, with a higher score indicating better health. 

*Depression.* The Geriatric Depression Scale–Short-Form (GDS-SF) was used. All items were on a “yes” or “no” scale. We calculated a sum score ranging from 0 to 15, with a higher score being suggestive of more depressive symptoms. The GDS-SF is reliable and valid across settings [57,58].

*Chronic Pain.* We measured chronic pain using the McGill Pain Questionnaire–Short-Form 2 (MPQ-SF-2) [59]. This measure has 22 items that measure experience of pain in the past week. Each item ranged from 0 (none) to 10 (worst possible). We calculated a total pain score which was average of all responses. The score ranged from 0 to 10, with a higher score indicating severe pain.

*Current Smoking Status.* Participants were asked whether they smoke. The exact question was: “How would you describe your cigarette smoking habits?”. Response options included: Still smoke, used to smoke, and never smoked. We combined the responses to have a dichotomous variable which indicated current smokers versus any other situation. 

### 3.5. Outcome Variables

*Drinking.* Participants reported if they drink alcohol. The item was: “Do you drink alcohol?”. This variable was operationalized as a dichotomous variable. 

*Alcohol Binge Drinking.* Participants who answered positively to the question of whether they drink alcohol were asked “How often did you have six or more drinks?”. Responses ranged from 0 to 6 which were for never, a few times a year, every few months, monthly, every few weeks, weekly, and daily. Due to having a very low number of individuals who had high levels of binge drinking in the sample, we categorized binge drinking as a dichotomous variable: 0 no alcohol binge drinking vs. 1 alcohol binge drinking.

### 3.6. Statistical Analysis

We used SPSS 23.0 for data analysis. To rule out collinearity between study variables, Pearson correlation test was utilized to estimate bivariate correlations between the study variables. We applied logistic regression models with drinking and binge drinking as the dependent variables, demographics, SES, living arrangement, marital status, smoking, and health status as the independent variables. As almost all participants had a health insurance, we did not include this variable in our multivariable models. In addition, we ruled out any collinearity between our independent variables including but not limited to living arrangement and marital status. From our models, we reported odds ratio (OR), standard error (SE), 95% confidence interval (95% CI), and *p* values. 

## 4. Results

### 4.1. Descriptive Statistics

Table 1 describes the study participants. Participants were all older adults (65+ years) with an average age of 74 years (SD = 6.7). Participants were mostly women (69.3%), were living alone (55.8%), and were not married (84.4%). Almost all participants (99.6%) had health insurance. From all participants, 23.8% and 8.7% reported drinking and binge drinking, respectively. 

From 160 women, 37 people (23.1%) and from 71 men, 18 people (25.4%) reported alcohol drinking (*p* > 0.05). In addition, from 160 women, 6 people (3.8%) and from 71 men, 12 people (19.7%) had binge drinking (*p* < 0.05). 

### 4.2. Bivariate Analysis

Table 2 shows the results of bivariate correlations between study variables. This table reports correlation coefficients (r) as well as p value levels. These correlations are based on Pearson correlation test. Age and gender but not SES indicators correlated with drinking and binge drinking. Living alone, CMC, smoking, and chronic pain but not depression and SRH correlated with drinking and binge drinking.

### 4.3. Multivariable Analysis

Table 3 shows the results of our logistic regression model for drinking as the outcome. This model shows that age (OR = 0.92, 95% CI = 0.86–0.98) but not gender was associated with odds of drinking. Neither educational attainment nor financial strain were associated with odds of drinking. At the same time, age (OR = 0.92, 95% CI = 0.86–0.98), living alone (OR = 3.09, 95% CI = 1.30–7.36), CMCs (marginal) (OR = 0.80, 95% CI = 0.63–1.01), smoking (marginal) (OR = 2.79, 95% CI = 0.96–8.12), and pain (OR = 1.14, 95% CI = 1.75–9.76), but not marital status and SRH were associated with odds of drinking.

Table 4 shows the results of our logistic regression model for binge drinking as the outcome. This model shows that age, educational attainment, financial strain, living alone, marital status, and SRH were not associated with odds of binge drinking. At the same time, gender (OR = 0.10, 95% CI = 0.03−0.35), CMCs (OR = 0.64, 95% CI = 0.43−0.96), smoking (OR = 4.12, 95% CI = 1.08−15.74), and pain (OR = 1.36, 95% CI = 1.00−1.85) were associated with odds of binge drinking.

## 5. Discussion

This study explored social, behavioral, and medical correlates of drinking and binge drinking in economically challenged African American older adults diagnosed with T2DM. Based on our study, age, living alone, smoking, CMC, and pain and were associated with odds of drinking. In addition, gender, smoking, CMC, and pain were associated with the odds of binge drinking. As most of our sample was males, this observation refers notably to male participants.

In line with the Minorities Diminished Returns (MDRs) theory [60,61,62,63], we did not observe strong effects of SES on drinking behaviors of our sample. This pattern is shown across health outcomes [64,65,66] and may be due to racism and discrimination that does not allow SES to turn into health outcomes [67,68,69,70,71,72,73]. Similarly, depression does not show many of the expected correlations in African Americans [74,75,76,77,78,79,80]. We also did not expect a link between depression and binge drinking in this study. In a study, number of psychiatric disorders such as anxiety and depression was associated with binge drinking in Africa American men [43]. However, we expected loneliness/living alone [36,37], pain [39], and CMC [34,35] to predict drinking and binge drinking in our sample.

We did not find any role of educational attainment or financial strain on drinking or binge drinking in African American older adults diagnosed with T2DM. Two recent studies show that in African American older adults, subjective but not objective SES impacts health and health behaviors. That means, it was financial difficulty but not educational attainment that impacted smoking, drinking, SRH, CMC, depression, pain, and % sick days. This meant that highly educated African American older adults were at the same risk of poor health, as well as smoking and drinking, as their less educated African American counterparts. 

The finding that SES indicators such as educational attainment have less than expected health effects on inner-city African American older adults is not new. Smaller or no protective effects of educational attainment and other objective SES resources are called MDRs [60,61,62,63,64]. These patterns are not innate, biologic, or related to populations’ culture. Instead, they are due to structural racism across institutions [63]. These MDRs apply to African Americans [60,61,62,63,64], Hispanics [64,81], and sexual minorities [82,83]. For Whites, the socially dominant group, higher educational attainment translates to better health and lower risk behaviors [22,23]. For Whites, the sky is the limit, as structural factors (e.g., racism) are not systemically blocking their outcomes.

It was unexpected to see that even financial difficulty did not impact drinking behaviors of our participants. Financial difficulty reflects lack of money, cash, expendable income, and liquid financial resources that can buffer stress and shape access to resources. Financial difficulty and financial strain have been shown to impact alcohol use, smoking, suicide, depression, self-rated health, chronic disease, and mortality [84,85,86,87,88,89,90,91]. These effects are repeatedly documented for the general population, as well as people with chronic disease [85,86,87,88,89,90,91]. Older adults and individuals who have T2DM, cancer, and heart disease are hit hard by financial difficulty [86,87,88,89,90,91]. All these studies collectively provided undeniable evidence on financial difficulty as one of the strongest social determinants of health [84,85,86,87,88,89,90,91]. Financial difficulty increases health problems through various ways. It is a specific type of stress [91]. It limits the choices that are available to maintain health [92] and increases health risk behaviors such as alcohol use and smoking [84]. It reduces ability to access a healthy diet [93] and access health care services when needed [92]. Considering all these effects together, we had expected financial difficulty to become another major constraint and increase risk behaviors of low SES African American older adults diagnosed with T2DM. Our hypothesis, however, was not supported by the data. 

The result highlights a need to address loneliness and pain when we want to promote T2DM management among the African American community. Reducing severity and chronicity of pain may remain a hopeful strategy to promote T2DM management in African American older adults who live in urban areas use alcohol or have binge drinking. African American older individuals diagnosed with T2DM who live alone, are younger, and experience severe pain are at most risk, and may need most of our attention. Despite pain is more common in women than men, and despite pain intensity is a predictor of binge drinking [94,95], it is men not women who have binge drinking, as shown in our study.

Our findings suggest that policies should also minimize the diminished returns of SES for African American older adults in urban settings. Policymakers should be aware that merely enhancing SES may not be enough to tackle some of the health disparities that affect the African American community, particularly those who have T2DM and other CMCs. Policies should go beyond health policy and help African American older adults mobilize their SES indicators and resources. Policy makers should also not expect too much from any policies that only enhances SES. Until racism and segregation continue as is, such economic policies that merely enhance access of populations to resources may result in less than expected effects. A real change may require may need more equal distribution of political power, and more effective discourse on racism, segregation, and historical injustice [96,97,98]. 

For several reasons, alcohol binge drinking is a major risk to the African American communities [9,10]. Predatory marketing, targeted advertisement, high availability of alcohol outlets, different patterns of initiation, combined with high stigma and mistrust towards the health care system all increase the vulnerability of this population to binging alcohol [11,12]. Such vulnerability is also in part due to low availability of treatment services in poor urban areas where the many African Americans live [11,12]. As a result of such vulnerability, a phenomenon called telescoping effect has been described that is defined as a rapid transition of substance use (e.g., alcohol use) in African Americans and other vulnerable groups from initiation to undesired outcomes [13,14]. As a result of this phenomenon, despite lower overall prevalence of alcohol use within this subgroup, the undesired health outcomes of alcohol and substance use are more common in the African American community, particularly those who are economically challenged [13,14]. 

Clinicians should be aware that African American older adults diagnosed with T2DM, and those with loneliness and pain are at an increased risk of binge drinking. As binge drinking is a barrier against successful T2DM management, there is a need to monitor drinking status of African Americans who have a painful condition and live alone. Pain management may also be related to drinking habits of African American older adults diagnosed with T2DM [99]. More research is needed to understand how reduction of pain can help with reducing binge drinking of this population. 

This study shows that age, living alone, CMC, smoking, and pain may inform programs that wish to screen binge drinking in African American older adults diagnosed with T2DM. In line with the MDR theory, SES does not seem to impact African American older adults diagnosed with T2DM’s drinking, which is probably due to the constrains imposed by racism and segregation as well as poor urban areas. It is unclear whether African American older adults diagnosed with T2DM use alcohol to cope with pain or loneliness or not.

There are several limitations to this study. A cross-sectional design of our study limits any causal inference in this study. All the variables were limited to the individual level. Important confounders such as history of psychiatric disorders were not included. The sample size was only modest, and the sample was skewed by a large proportion being females (70%) who are less likely to drink and drink much less than the male participants. In this sample, only 55 participants (24%) reported drinking alcohol which heavily decreases the robustness of the results obtained. Given these limitations, the results should be interpreted with caution and the findings should be considered as suggestive and preliminary. 

## 6. Conclusions

In economically challenged African American older adults diagnosed with T2DM, age, living alone, smoking, CMC, and pain and were associated with odds of drinking, and gender, smoking, CMC, and pain were associated with the odds of binge drinking. These factors may help clinicians and program developers to leverage drinking prevention as a way to improve T2DM outcomes in African American older adults. 

## Figures and Tables

**Table 1:** Descriptive Statistics.

Characteristics		
	*Mean*	*SD*
Age (Years)	74.12	6.66
Educational Attainment (Years)	12.59	2.49
Financial Strain	8.07	4.68
Comorbid Medical Conditions (CMC)	3.64	1.82
Self-Rated Health (SRH)	3.13	0.92
Depressive Symptoms	2.19	2.57
Chronic Pain	1.73	2.02
	*n*	*%*
Gender		
Men	71	30.7
Women	160	69.3
Age		
Older Old	165	71.4
Younger Old	66	28.6
Family Type		
Others	195	84.4
Married	36	15.6
Living Arrangement (Living Alone)		
No	102	44.2
Yes	129	55.8
Health Insurance (Any)		
No	1	0.4
Yes	230	99.6
Current Smoking Status		
No	209	90.5
Yes	22	9.5
Drinking Alcohol		
No	176	76.2
Yes	55	23.8
Binge Drinking		
No	211	91.3
Yes	20	8.7

**Table 2:** Bivariate correlations.

	1	2	3	4	5	6	7	8	9	10	11	12	13
1 Gender (Female)	1.00	0.02	0.11	0.02	−0.23 **	0.09	0.15 *	0.16 *	−0.01	0.08	−0.14 *	−0.02	−0.26 **
2 Age (Years)		1.00	−0.19 **	0.01	−0.14 *	0.16 *	0.03	−0.10	−0.01	−0.07	−0.23 **	−0.19 **	−0.11
3 Educational Attainment (Years)			1.00	−0.07	0.11	−0.05	−0.02	0.11	−0.09	0.05	0.00	0.07	−0.01
4 Financial Difficulty				1.00	−0.01	0.12	0.23 **	0.18 **	0.22 **	0.31 **	0.12	0.14 *	0.07
5 Family Type (Married)					1.00	−0.41 **	0.07	−0.03	0.05	−0.03	0.10	−0.04	0.00
6 Living Arrangement (Living Alone)						1.00	0.09	0.02	0.10	0.19 **	−0.07	0.17 **	0.09
7 Chronic Medical Conditions (CMC)							1.00	0.31 **	0.35 **	0.49 **	0.00	−0.04	−0.08
8 Self-Rated Health (SRH)								1.00	0.30 **	0.38 **	0.05	0.05	0.08
9 Depression									1.00	0.39 **	0.11	−0.01	0.06
10 Chronic Pain										1.00	0.02	0.20 **	0.11
11 Smoking (Current)											1.00	0.17 *	0.22 **
12 Drinking Alcohol												1.00	0.55 **
13 Binge Drinking													1.00

* *p* < 0.05, ** *p* < 0.01.

**Table 3:** Multivariable logistic regression model with drinking as the outcome.

	B	S.E.	Exp(B)	95% CI for Exp(B)	Sig.
Gender (Female)	−0.19	0.39	0.82	0.38–1.79	0.623
Age (Years)	−0.08	0.03	0.92	0.86−0.98	0.010
Educational Attainment (Years)	0.05	0.08	1.05	0.90−1.23	0.515
Financial difficulty	0.05	0.04	1.05	0.97−1.13	0.198
Marital (Married)	0.20	0.60	1.22	0.38−3.99	0.736
Living Arrangement (living alone)	1.13	0.44	3.09	1.30−7.36	0.011
Current Smoking	1.02	0.55	2.79	0.96−8.12	0.061
Chronic medical conditions (CMC)	−0.22	0.12	0.80	0.63−1.01	0.065
Self-rated health (SRH)	0.05	0.21	1.05	0.69−1.60	0.824
Sick days	0.44	0.19	1.56	1.07−2.27	0.022
Depression	−0.10	0.09	0.91	0.77−1.07	0.259
Chronic Pain	0.35	0.11	1.41	1.14−1.75	0.002

**Table 4:** Multivariable logistic regression model with binge drinking as the outcome.

	B	S.E.	Exp(B)	95% CI for Exp(B)	Sig.
Gender (Female)	−2.28	0.63	0.10	0.03−0.35	0.000
Age (Years)	−0.08	0.05	0.93	0.84−1.03	0.147
Educational Attainment (Years)	0.07	0.11	1.07	0.86−1.34	0.532
Financial difficulty	0.01	0.06	1.01	0.90−1.12	0.896
Marital (Married)	−0.21	0.99	0.81	0.12−5.69	0.833
Living Arrangement (living alone)	1.23	0.78	3.42	0.74−15.80	0.115
Smoking	1.41	0.68	4.12	1.08−15.74	0.039
Chronic medical conditions (CMC)	−0.44	0.21	0.64	0.43−0.96	0.032
Self-rated health (SRH)	0.44	0.31	1.56	0.84−2.88	0.157
Sick days	0.07	0.28	1.08	0.62−1.85	0.791
Depression	0.02	0.12	1.02	0.81−1.30	0.843
Chronic Pain	0.31	0.16	1.36	1.00−1.85	0.049
